# Single-cell time-lapse analysis of depletion of the universally conserved essential protein YgjD

**DOI:** 10.1186/1471-2180-11-118

**Published:** 2011-05-27

**Authors:** Tobias Bergmiller, Rafael Peña-Miller, Alexander Boehm, Martin Ackermann

**Affiliations:** 1 Department of Environmental Sciences, ETH Zurich, Switzerland, and Department of Environmental Microbiology, Eawag, Switzerland; 2 Biosciences, University of Exeter, Exeter, UK; 3 Institut für Molekulare Infektionsbiologie, University of Wuerzburg, Germany

## Abstract

**Background:**

The essential *Escherichia coli *gene *ygjD *belongs to a universally conserved group of genes whose function has been the focus of a number of recent studies. Here, we put *ygjD *under control of an inducible promoter, and used time-lapse microscopy and single cell analysis to investigate the phenotypic consequences of the depletion of YgjD protein from growing cells.

**Results:**

We show that loss of YgjD leads to a marked decrease in cell size and termination of cell division. The transition towards smaller size occurs in a controlled manner: cell elongation and cell division remain coupled, but cell size at division decreases. We also find evidence that depletion of YgjD leads to the synthesis of the intracellular signaling molecule (p)ppGpp, inducing a cellular reaction resembling the stringent response. Concomitant deletion of the *relA *and *spoT *genes - leading to a strain that is uncapable of synthesizing (p)ppGpp - abrogates the decrease in cell size, but does not prevent termination of cell division upon YgjD depletion.

**Conclusions:**

Depletion of YgjD protein from growing cells leads to a decrease in cell size that is contingent on (p)ppGpp, and to a termination of cell division. The combination of single-cell timelapse microscopy and statistical analysis can give detailed insights into the phenotypic consequences of the loss of essential genes, and can thus serve as a new tool to study the function of essential genes.

## Background

Genes that are highly conserved between different types of organisms code for important biological functions and are therefore usually well studied and described. One group of conserved genes whose function has remained enigmatic until recently is the Kae1(OSGEP)/YgjD family. Genes from this family occur in almost all bacterial, archaeal and eukaryotic genomes. The gene family consists of two groups: one group, GCP1/OSGEPL/Qri7, is of bacterial origin, the other, GCP2/OSGEP/Kae, is supposed to originate from archaea [1]. *In Escherichia coli*, Kae1/YgjD is essential for viability [2,3]; in *Arabidopsis thaliana *and *Saccharomyces cerevisia*, deletion mutants exhibit deleterious phenotypes [4-6].

A biochemical activity for YgjD has recently been described: as already suggested by [7], Srinivasan and colleagues [8] showed that Kae1/YgjD protein (of *Saccharomyces cerevisiae *and *Escherichia coli*, respectively) is required to add a threonyl carbamoyl adenosine (t^6^A) modification to a subset of tranfer-RNAs that recognize codons with an adenin at the first position. Transfer-RNAs undergo complex modifications and maturation steps [9] required for translational fidelity [10-12]. Mutations in these modification pathways can be lethal or cause severe defects [13-15], and the involved genes are highly conserved in different organisms [14-16].

Because ygjD is essential, it is not possible to delete the gene and study the phenotypic consequences. As an alternative, one can put the gene under control of an inducible promoter, and investigate the consequence of turning off its expression, and thereby depleting the YgjD protein. Our aim here is to get insights into the morphological changes that come about when the YgjD protein is depleted from growing *Escherichia coli *cells. In two studies ([3] and [17]), the authors have noticed an effect on cell size in YgjD depletion strains, suggesting a role of YgjD for cell division and/or cellular elongation. However, while Katz *et al*. observed shorter cells under YgjD depletion conditions, Handford *et al*. observed a mixed population of elongated and short cells. The reason for this discrepancy remained unclear but could be based on the different genetic background of the *E. coli *strains (MC4100 versus MG1655). Altered cell size upon YgjD depletion could be based on changes in cell division timing or the cellular elongation rate, or on a combination of these two effects. To distinguish between these possibilities and to clarify the role of YgjD for cell size we used single cell resolution time-lapse microscopy of growing microcolonies.

We constructed a conditional lethal *ygjD *mutant, and investigated the consequences of depletion of the YgjD protein with high temporal resolution at the single-cell level. Similarly to ([3,6,17]) we put the expression of *ygjD *under control of a promoter that is inducible by the sugar L-arabinose. The resulting strain can be grown normally in presence of L-arabinose, but ceases to grow in absence of L-arabinose and presence of glucose. Then, single bacterial cells are placed on a nutritious agar surface lacking the inducer and are observed with time lapse microscopy.

We used the cell tracking software "Schnitzcell"[18] to analyze images from the time-lapse microscopy experiments. This software identifies cells and tracks them across images from consecutive time points. It keeps track of cell division events and of relatedness of cells (e.g., it can relate each cell to the other cell that emerged from the same division). The software also extracts information about cell size and fluorescence intensity. The resulting dataset can be used to reconstruct the lineage of the clonal microcolony, and to plot phenotypic information like cell size and fluorescence intensity on this lineage. We used derivatives of these parameters (cell elongation rate and interval between divisions) to describe and analyze the effects of YgjD depletion.

We find that depletion of YgjD changes the balance between cell growth and cell division, indicating a disturbance in cell size homeostasis. Experiments with *Escherichia coli *and *Salmonella thyphimurium *have shown a high degree of cell size homeostasis, or balanced growth [19]: under steady state conditions, cells have a constant cell size, indicating that the rate by which cells elongate and the interdivision intervals are coupled - cells that grow slower will initiate cell division later, and thus reach a goal cell size despite their slower growth. Under conditions of YgjD depletion, cell elongation slowed down while the interval between cell divisions remained constant. As a consequence, cell size steadily decreased over consecutive divisions, until a minimal size was reached and cell division stopped. These cellular changes are specific: they differ from the consequences of the depletion of three other essential genes we analyzed, and of the exposure to two antibiotics that inhibit translation.

Using a statistical approach, we show that this growth transition occurs in a controlled manner: cell elongation rates and interdivision times were negatively correlated on the level of single cells, suggesting that these two physiological parameters remain coupled during the growth transition. Genetic experiments indicated that this change in cell size homeostasis involves production of the alarmone (p)ppGpp (guanosine-penta/tetra-phosphate), a signaling compound that is a key player of a cellular response to amino acid starvation known as stringent response.

## Results and Discussion

**Figure 1:**
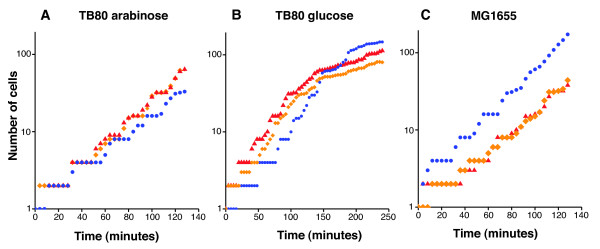
***ygjD*-expression determines patterns of growth**. Each panel depicts data of cell numbers versus time from three independent experiments; each experiment is based on a microcolony that was initiated with a single cell, and followed over about six to seven divisions. **A**) TB80 (P_ara_-*ygjD*) grown in presence of 0.1% L-arabinose. **B**). TB80 (P_ara_-*ygjD*) grown in presence of 0.4% glucose. Note that the growth rate decreased after about 150 minutes. **C**) MG1655 (*E. coli *wild type) grown in LB medium with additional 0.4% glucose. Growth rates are similar to panel A, indicating that the induction of *ygjD*-expression in TB80 (panel **A**) lead to growth rates that are similar to wild type *E. coli*.

Our rationale here is that we can get insights into the biological role of YgjD by following the cellular response of its depletion on the single cell level and with high temporal resolution. We diluted cultures of the conditional lethal P_*ara*_*-ygjD *mutant TB80 onto pads of solid LB medium that either contained L-arabinose (inducing *ygjD *expression) or D-glucose (repressing *ygjD *expression) and used time-lapse microscopy to follow single cells growing into microcolonies, taking an image every 2 or 4 minutes. The images were analyzed with the software "Schnitzcell" [18]. The growth rate and cellular morphology of the P_*ara*_-*ygjD *strain grown in the presence of L-arabinose was similar to the wild type grown under the same conditions (Figure 1a and 1c, and Additional file 1 - movie 1 and Additional file 2 - movie 2).

A shift of the P_*ara*_-*ygjD *strain to glucose lead to the depletion of YgjD. This depletion is based on two effects. First, transcription of *ygjD *stops after the shift to glucose. Residual L-arabinose that remains in the cells from growth under permissive conditions is rapidly metabolized. Lack of L-arabinose turns the transcriptional activator (AraC) of the P_ara _promoter into a transcription repressor. In addition, glucose metabolism causes depletion of the cellular co-inducer cyclic AMP. Together these effects lead to effective repression of *ygjD *transcription in TB80. After termination of *de novo ygjD *mRNA synthesis the amount of YgjD in each cell declines, because the mRNA and the protein are diluted through cell division, and degraded by cellular nucleases and proteases, respectively [20]. The rapid cessation of transcription from P_ara _after the shift to glucose was evident in control experiments with a strain that expressed the green fluorescent protein (GFP) from the arabinose promoter (Additional file 3 - Figure S1)

### YgjD depletion leads to a change in cell size homeostasis

**Figure 2:**
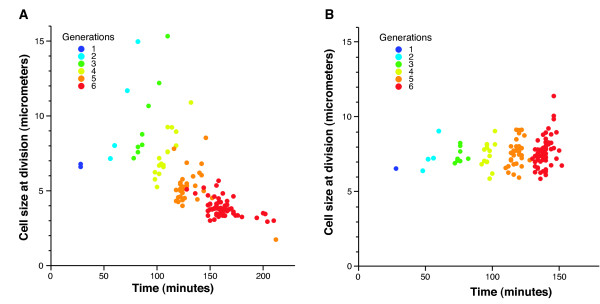
**Depletion of YgjD lead to a change in cell size homeostasis**. The figure is based on data from one microcolony of TB80 (P_ara_-*ygjD*) depleted for YgjD (**A; **also see Additional File 4 - movie 3) and *E. coli *wildtype MG1655 (**B**; also see Additional File 2 - movie 2). Each point represents information about one cell, and the color of the point indicates which generation this cell belongs to (for a definition of 'generation' see main text). **A**) Changes in cell size during YgjD depletion. Cell size at division decreases continuously during the depletion experiment; **B**) Growth characteristics of MG1655 on single cell level. MG1655 exhibits a nearly constant cell size at division, and a slight increase of growth rate over consecutive divisions.

Time-lapse microscopy revealed that YgjD depletion lead to a gradual phenotypic transition in division and growth. Single cells that were transferred from permissive conditions to solid pads of LB medium with glucose first continued to divide regularly, forming microcolonies in which the number of cells initially increased exponentially. Then, after about four divisions, cell division slowed down and stopped (Figure 1b and Additional file 4 - movie 3). Analysis of the time-lapse images (Additional file 4 - movie 3) showed that, during this transition, cells size decreased (Figure 2a). This indicates a disturbance in of cell size homeostasis [19] - that cells divide before their cell size doubled.

We used elongation rates of single cells and the time interval between two divisions to analyze the change in cell size homeostasis during YgjD depletion. Since we were interested in how these parameters changed during depletion, we separated data from different cell generations of the depletion process. The first cell that is founding a microcolony is generation 0; this cell divides into two cells of generation 1, which divide into four cells of generation 2, and so on (also see Additional File 5 - Figure S2). To avoid comparisons between cells that are in different phases of their cell cycle, we only used cell size measurements (and later fluorescence intensities) of cells immediately before division. Also, to avoid incomplete and biased sampling, we removed data from above generation 6.

**Figure 3:**
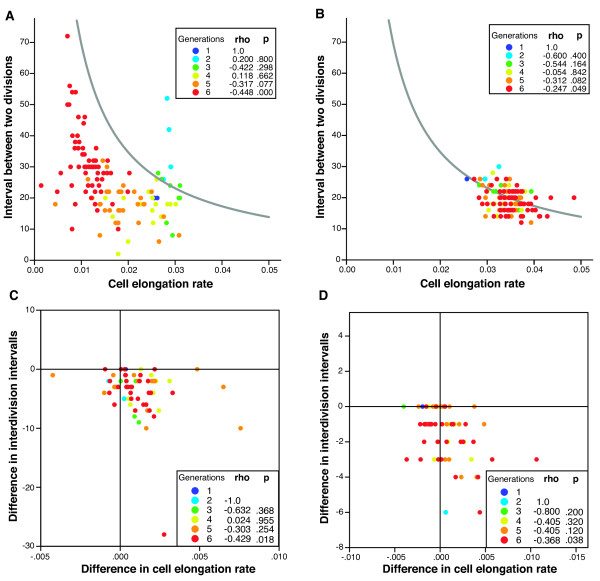
**Cell elongation rate and the interval between two divisions are coupled during YgjD depletion**. The contour line depicts all combinations of cell elongation rate and interval between divisions that correspond to a cell size doubling before division. Points below the contour line corresponds to cells that divide before they double in size, and whose size thus steadily declines. **A) **The relationship between the cell elongation rate and the interval between two divisions during YgjD depletion (**Movie 2, additional files**), and **B) **for MG1655 (**Movie 3, additional files**). For YgjD depletion, cell elongation rate starts to decrease from generation 3 on. However, this decrease in cell elongation rate is initially not compensated for by an increase in the interval between two divisions. Points below the contour line correspond to cells that divide before they double in size, and whose size thus steadily declines. The inset lists the result of a non-parametric correlation analysis between 'cell elongation rate' and 'time to division', performed separately for every generation. A negative correlation indicates coupling of the interval between division and the cell elongation rate. For MG1655, the majority of cells cluster around the contour line. **C**) and **D**) show the result of the independent contrast correlation analysis for YgjD depletion in TB80, and MG1655 growth. Each point depicts the difference (residual) between two sister cells in the cell elongation rate (horizontal axis) and in the interval between cell divisions (vertical axis). Cells that have a higher elongation rate than their sister tend to have a shorter interval between divisions. The inset lists the result of a non-parametric correlation analysis between 'difference in cell elongation rate' and 'difference in interval between two divisions', performed separately for every generation. Again, negative correlation indicates coupling of the interval between division and the cell elongation rate.

This analysis revealed that the small size of cells depleted for YgjD was a consequence of two effects: first, the rate of elongation (cell length increase over time) decreased (Figure 3a). Second, cells did not respond to the decrease in elongation rate by adjusting the frequency at which they divided; the interval between two cell divisions remained initially constant. As a direct consequence, cell length at division decreased continuously (Figure 2a).

The phenotype induced by YgjD depletion was specific, and depletions of other essential genes lead to different cellular morphologies. We analyzed time-lapse images of the depletion of three other essential genes (*dnaT*, *fldA *and *ffh*). Depletion of each protein resulted in cellular phenotypes that were different from each other and from YgjD when depleted (Additional file 6 - Figure S3; also see Additional Files 7, 8 and 9 - movies 4, 5 and 6). Also, the effects of YgjD depletion were different from the consequences of exposure to two antibiotics that we tested: we followed wildtype *E. coli *cells exposed to the translational inhibitors kanamycin and chloramphenicol at minimum inhibitory concentration (2.5 μg/ml for chloramphenicol, 5 μg/ml for kanamycin), and observed no decrease in cell size (Additional file 10 - Figure S4, and Additional Files 11 and 12 - movies 7 and 8).

For reference, we also analyzed images of growing microcolonies of wildtype *E. coli *MG1655 cells on LB medium supplemented with glucose. This experiments confirmed cell size homeostasis, as expected from normally growing cell: cells divided close to the moment when they had doubled their size, and small fluctuations in cell elongation rates were compensated for by adjustments in the time of cell division (Figure 2b and 3b).

### The transition towards smaller cell size is controlled

What kind of disturbance of cell size homeostasis is induced by depletion of YgjD? We considered two possibilities. First, it is possible that the control that couples cell division to cell size is lost, so that cells divide in an uncontrolled way, irrespective of their size. Second, it is conceivable that cell division remains coupled to cell size, but the target size that a cell needs to reach before initiating division decreases over time.

If the decrease in cell size is the result of a controlled transition towards smaller cells, one would expect that, during the transition, the cell elongation rate and the timing of cell division would still be linked, but that this link would change quantitatively over time. In fact this is what we observed when we analyzed each generation of cells during the depletion process separately (inserts Figure 3a and 3b). Within a given generation the time interval between divisions and the rate by which a cell elongated was negatively correlated: cells that grew faster than the average of their generation tended to initiate division more quickly; cells that grew more slowly initiated division later. This suggests that cell growth and the timing of cell division are still linked within each generation in the depletion process, but that this link changes quantitatively over successive generations.

This analysis has, however, an important limitation: cells within a given generation are not independent from each other. Some of these cells are more closely related, because they derive from the same mother or grandmother. This can lead to spurious correlations between traits; in our case, this effect could lead to artificial correlations between cell elongation rates and interdivision intervals. This problem of relatedness in lineage trees is known from phylogenetic studies, where it is referred to as phylogenetic dependence [21]. In the context of phylogenetic studies, these dependencies can be resolved by analyzing differences between independent *pairs *of species, rather than calculating correlations on the basis of the whole phylogenetic lineage [21].

We used a variation of this approach to get an unbiased view on the relationship between cell growth and the timing of cell division: for each generation, we analyzed pairs of cells emerging from the same cell division, and calculated the difference in growth rates and in the time to division for each pair. We refer to two cells emerging from the same division as 'sisters' (thereby ignoring that these two cells have cell poles of different ages, [22,23]). The differences for all sister pairs represent independent data points, and we can use them to calculate the correlation between cell growth and time to division in an unbiased way.

The independent contrast analysis confirmed our earlier conclusions: comparing each cell to its sister cell, we found that cells that grew slower than their sisters also displayed a longer interval between cell divisions, while faster growing cells divided earlier. This manifests as a negative correlation between the difference in cell elongation rate and the difference in interdivision intervals between two sisters (inserts Figure 3c and 3d; see also Additional File 13 - Figure S5). This is consistent with the interpretation that, during YgjD depletion, the timing of cell division remained coupled to a given cell size - and that the target cell size declined.

The transition to decreased cell size is reminiscent of morphological changes that occur during the 'stringent response' [24,25], a stress adaptation program that is elicited when cells encounter amino-acid or carbon-starvation [26]. The stringent response is induced by accumulation of the 'alarmone' guanosine tetra/penta phosphate ((p)ppGpp), e.g. in response to low concentrations of amino-acylated tRNAs [26]. We thus wanted to investigate this possible link to (p)ppGpp signaling more closely, and asked whether the changes in cell homeostastis upon YgjD depletion are mediated through (p)ppGpp.

### Changes in cell size homeostastis are mediated through ppGpp

**Figure 4:**
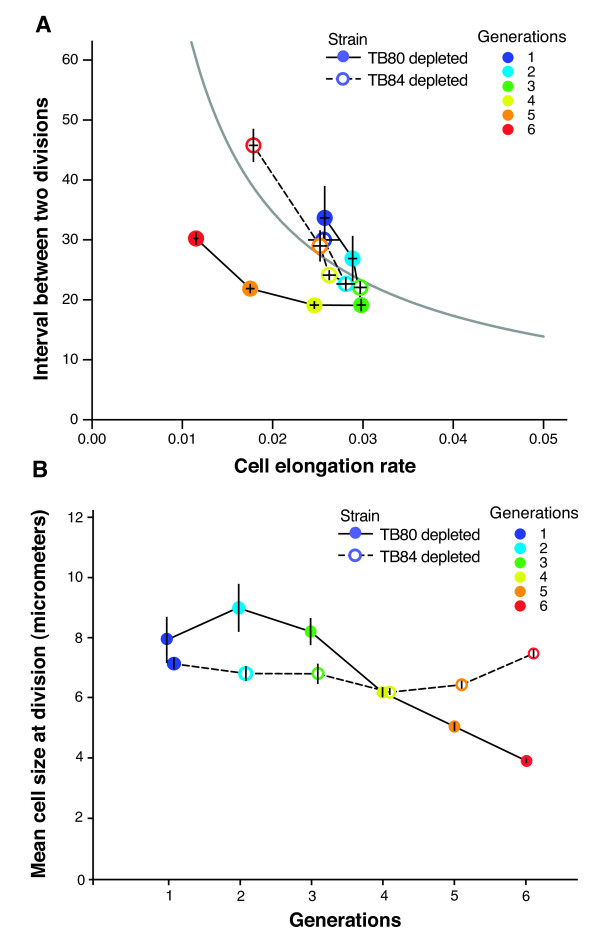
**The change in cell size homeostasis in response to YgjD depletion depends on (p)ppGpp**. **A) **Changes in cell elongation rate and the interval between two divisions during YgjD depletion, for TB80 (ppGpp^+^) and TB84 (ppGpp^0^). For each strain, means and standard errors of three independent experiments are shown. In TB80, cell elongation rate starts to decrease after generation 3, and cells divide before they double in size. In TB84, cell division occurs close to the moment of cell size doubling (the means are close to the contour line of constant cell size). **B) **Change of mean cell size during YgjD depletion, for and TB80 (ppGpp+) and TB84 (ppGpp-). In TB80, cell size starts to decrease after generation 3, as a consequence of cell division that occurs before cells double in size (see panel **A**). In TB84, cell size decreases only slightly, and then increases after generation 5, when cells divide after the moment of cell doubling (see panel **A**).

We constructed a strain, TB84, that is deficient in (p)ppGpp synthesis ((p)pGpp^0^), due to deletions of *relA *and *spoT *[26,27], and in which expression of *ygjD *was again under control of P_ara_. We followed growing microcolonies of TB84 as described above and found that the consequences of YgjD depletion were profoundly different: cell elongation rate decreased during the YgjD depletion process as for the *relA*^+ ^*spoT*^+ ^strain TB80 (Figure 4a). In contrast to what we observed with this (p)ppGpp^+ ^strain, the decrease in elongation rate was compensated for by an increase in the time interval between two divisions (Additional file 14 - movie 9, and Figure 4a). As a consequence, cell size at division was not reduced, and the final cell length of depleted (p)ppGpp^0 ^cells (TB84) was on average twice that of depleted (p)ppGpp^+ ^cells (TB80) (Figure 4b). This is reminiscent of the elongated cells found in populations of cells depleted for YgjD by Handford and colleagues [3].

This suggests that the changes in cell size in response to YgjD depletion are mediated through the alarmone (p)ppGpp; an alternative explanation is that the absence of (p)ppGpp leads to cell elongation (as has been previously reported [27]), and that this elongation compensates indirectly for reductive fission upon YgjD depletion. Importantly, TB84 cells still ceased cell division (Additional file 15 - Figure S6). Thus, *ygjD *is still essential even in the absence of (p)ppGpp, and termination of cell division is not solely a consequence of a diminished cellular growth rate.

**Figure 5:**
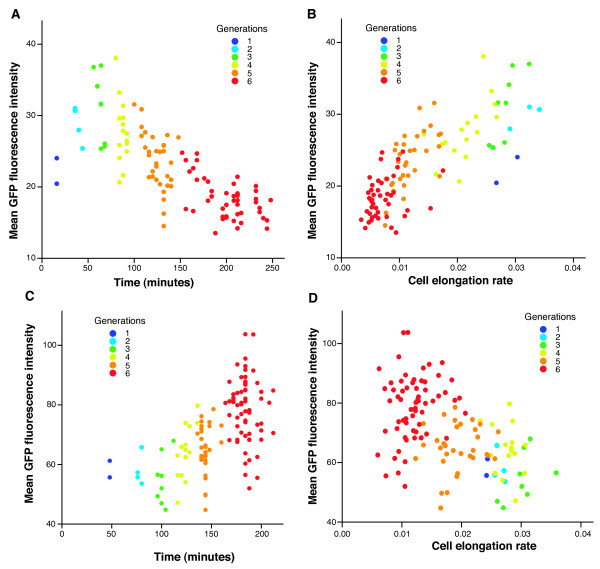
**Expression of P**_**apt **_**and P**_**rsd **_**during YgjD depletion**. Single cell measurements of cell elongation rate and GFP fluorescence of two strains with transcriptional reporters for P_apt _(**A and B**) and P_rsd _(**C and D**). Each point represents a measurement for a single cell. In both strains, cell elongation rate decreased with increasing generations during YgjD depletion as shown in Figures 1B and 2A. **A) **and **B) **P_apt _is repressed by (p)ppGpp; its expression decreases during YgjD depletion, and decreases steadily with decreasing cell elongation rate. **C) **and **D) **P_rsd _is induced by (p)ppGpp; its expression increases during YgjD depletion, and steadily increases with decreasing cell elongation rate.

To further test the idea that *ygjD *depletion triggers (p)ppGpp synthesis we measured, on a single cell level during YgjD depletion, the activity of two promoters known to respond to the intracellular level of (p)ppGpp: P_apt _is repressed by (p)ppGpp, while P_rsd _is induced by (p)ppGpp [28]. We transformed TB84 with plasmids carrying transcriptional promoter-*gfp *fusions [29] encoding P_apt_-*gfp *and P_rsd_-*gfp*, and measured gene expression from these promoters as fluorescence intensity over consecutive cell divisions. The level of GFP expression steadily decreased in the strains where *gfp *was controlled by P_apt _(Figure 5a), and steadily increased when controlled by P_rsd _(Figure 5c). Furthermore, this change in fluorescence was tightly linked to the rate by which cells elongated (Figure 5b and 5d). When the same strains were grown on L-arabinose containing medium no consistent changes of fluorescence could be observed (Additional file 16 - Figure S7). These observations are consistent with the scenario that YgjD depletion induces (p)ppGpp synthesis, and thus influences promoters whose expression depends on the levels of (p)ppGpp.

Single cell analysis indicated that, in the cells depleted for YgjD, there is a link between decreased cell elongation rate and (p)ppGpp levels. Using independent comparisons between sister cells in the microcolonies undergoing YjgD depletion, we found that if a cell had a lower elongation rate than its sister, it also tended to have lower levels of GFP expressed from P_apt _(details not shown; for P_rsd_-*gfp*, this pattern was not observed). These data support the idea that the link between (p)ppGpp levels and the cell elongation rate is direct; for example, it is possible that high levels of (p)ppGpp *cause *low elongation rates [30].

Our results suggest further that YgjD depletion has two (possibly linked) effects: first, depletion triggers (p)ppGpp synthesis. Second, it leads to termination of cell division. To gain insights in which phase of the cell cycle YgjD-depleted cells are arrested we visualized the DNA-content of individual cells with DNA-staining and subsequent fluorescence microscopy (Additional File 17 - Figure S8). After YgjD depletion in (p)ppGpp^+ ^cells (TB80), DNA was localized at midcell and filled large areas of the cell (Additional File 17 - Figure S8 b), possibly indicating that cells were unable to carry out additional cell divisions due to "nucleoid occlusion" [31]. This mechanism prevents premature cell division before chromosomes have been distributed to opposite cell halves. However, termination of cell division also manifests in a (p)ppGpp^0 ^strain (Additional File 17 - Figure S8 c): depleted cells were elongated, and only a small fraction of the cell volume was filled with DNA. Thus, in the (p)ppGpp^0 ^background, nucleoid occlusion alone cannot be responsible for termination of cell division. The elongated phenotype of YgjD depleted (p)ppGpp^0 ^cells resembles filamentous cells blocked in cell division. However, since abrogating cell division is not inhibiting DNA replication or DNA segregation [32] it appears unlikely that YgjD directly affects cell division.

## Conclusions

Our results show that single cell experiments coupled with statistical analysis can uncover phenotypic transitions that come about when an essential gene is depleted. We captured phenotypic changes with high temporal resolution across several cell generations. Cell tracking techniques allowed us to build lineages of cells, and to analyze correlations between phenotypic traits at the level of sister cells emerging from the same division. This information can be used to describe growth transitions on the cellular level.

We found that YgjD depletion has two, possibly linked, effects: a decrease in cell size that is accompanied by accumulation of (p)ppGpp, and the arrest of cell division. The involvement of (p)ppGpp in the alteration of cell size homeostasis under YgjD depletion conditions might explain the discrepancies between two studies ([3] and [17]) that observed opposite effects on cell size upon YgjD depletion. Katz et al. [17] used a *relA*^+ ^*spoT*^+ ^strain that is very similar to the ppGpp^+ ^strain TB80 used here, and - consistent with our findings - observed shorter cells upon YgjD depletion. In contrast the MC4100 derivative that was used by Handford and colleagues [3] carries a *relA1 *allele. This allele is known to cause reduced cellular (p)ppGpp levels under certain growth conditions [26,33]. Thus, their finding of elongated cells upon YgjD depletion might be similar to what we observed with the ppGpp^0 ^strain TB84.

Our observations seem consistent with the finding that *ygjD *is involved in forming transfer-RNA base modifications [8]. Thus, depletion of YgjD protein leads to a pool of un- or undermodified transfer-RNAs (as described by [8]), possibly resulting in non-optimal interactions between transfer-RNAs and mRNA inside the ribosome. This could potentially elicit a stringent-response like program (governed by (p)ppGpp release) and explain the phenotypic consequences of YgjD depletion that we observed. Non-optimal interactions between non-modified tRNAs and mRNA could be similar to the effects caused by ribosomes that are stalled on "hungry" codons: these codons are unsuccessfully trying to pair with either rare transfer-RNAs or transfer-RNAs that are non-aminoacylated due to amino-acid limitation. Hungry codons can provoke the production of aberrant proteins by frame shifts, slides of the translational machinery or incorporation of noncognate transfer-RNAs [34,35]. This might also explain the slow onset of the consequences of YgjD depletion: accumulation of aberrant proteins would slowly increase over time and reach a level where several cellular processes might be affected simultaneously.

Although the biochemical activity of YgjD has been described [8], the cellular functions of YgjD are not completely resolved. It will be interesting to ask how the proteins in the YgjD/YeaZ/YjeE complex [3] of *Escherichia coli *are interacting to fulfill their functions, and to ask whether YgjD is involved in other cellular processes or responding to environmental cues. Single-cell observations of YgjD depletion experiments might be helpful to generate and test hypotheses about the essential role of this protein, and to help explain why it is so widely conserved.

## Methods

### Bacterial strains and growth medium

P1 transduction and TSS transformation were performed as described elsewhere [36,37]. Strain DY330 as well as strains harboring the plasmid pCP20 [38] were grown at 32°. All other strains were grown at 37°. To grow TB80 and TB84 under permissive conditions, we used LB medium (Sigma) supplemented with 0.1% (batch culture) or 0.01% (before time-lapse microscopy) L-arabinose (Sigma). LB agar (1.5% agar) was from Sigma, and used for preparing agar plates and agar pads for time-lapse microscopy.

### Strain construction

Strains containing more than one knockout or marker were generated by sequential P1-transductions. Resistance markers were removed by Flp recombinase mediated site-specific recombination [39]. To control expression of *ygjD*, we constructed a conditional mutant with a second copy of the promoter of the *araBAD *operon in front of the native chromosomal locus of *ygjD *by directly inserting a P_ara_-construct in front of *ygjD*, as described previously [40]. Removal of L-arabinose and addition of glucose allows tight repression of target genes under control of P_ara _[40,41].

We closely followed the description of [40] and first constructed a strain in which a kanamycin resistance cassette is linked to the promoter of *araBAD*. We inserted such a kanamycin marker downstream of araC with the following primers:

5'_araC_yabI_insert

AATCAGACAATTGACGGCTTGACGGAGTAGCATAGGGTTTTGTGTAGGCTGGAGCTGCTTC;

3'_araC_yabI_insert

GCATAATGTGCCTGTCAAATGGACGAAGCAGGGATTCTGCCATATGAATA

TCCTCCTTAGTTCCTAT.

The insertion was done in DY330 following the protocol described by [42], verified by PCR and moved to MG1655 by P1 transduction, thus generating TB55.

TB55 was subsequently used to generate a PCR product that spanned the kanamycin cassette adjacent to *araC*, *araC*, the full intergenic region between *araC *and *araB*, and 42 basepairs at the 5' and 3' -prime ends that were homologous to the upstream and 5'-coding region of *ygjD*, *dnaT *,*fldA *or *ffh*. The sequence of the primers was

ygjD_insert5' AGTTTTACATCAACCCGCATTGGTCCTACACTGCGCGGTAATAATGTGCCTGTCAAATGGACG

ygjD_insert3' GCCGGTTTCATCGCAGGAAGTTTCAATACCCAGTACACGCATCGTTTCACTCCATCCAAAAAA

dnaT_insert5' TCCGTGTGTTACTATAAAAGTTATCTCCCTTCTCGTTCATCGAATGTGCC

TGTCAAATGGACG

dnaTC_insert3' GTCAATACCAACGACGTCCGGGGTCAAAACTCTGGAAGACATCGTTTCAC

TCCATCCAAAAAA

ffh_insert5'

GACGCCTTCATGTTATACTGCGGCAAAATACTGATGATGTGTAATGTGCC

TGTCAAATGGACG

ffh_insert3' GCGCAGCGTGCGCGACAAACGATCGGTTAAATTATCAAACATCGTTTCAC

TCCATCCAAAAAA

fldA_insert5' TGCCTTTATCCGTGGGCAATTTTCCACCCCCATTTCAATAAGAATGTGCC

TGTCAAATGGACG

fldA_insert3'

ATTACCGGTGTCGCTGCCGAAAAAGATGCCAGTGATAGCCATCGTTTCAC

TCCATCCAAAAAA

DY330 cells were grown in LB medium supplemented with 0.2% L-arabinose and made electro- and recombination competent [42], and electroporated with the above described PCR product. After electroporation, cells were transferred to LB medium containing 0.1% L-arabinose and incubated at 32° for 1.5 hours prior to plating on LB plates agar containing L-arabinose (0.1%) and kanamycin (50 μg/ml, Sigma). Clones were checked on LB agar plates supplemented with 0.4% glucose to confirm that they were unable to grow in the presence of glucose.

The promoter fusions and the adjacent *araC *gene were verified by sequencing with the following primers:

araC_FW GCTACTCCGTCAAGCCGTCA;

ygjD_RW GGCAATTGGTCTGGGGAGCA.

dnaTC_RW AGAGTTGATCGTCCAGAGCG

ffh_RW ATTTTGACGAACTCCTGCCC

fldA_RW CGAGAGTCGGGAAGAAGTCA

The constructs were then moved by P1 transduction into MG1655. To construct TB80 the kanamycin cassette was removed with pCP20.

The knockout Δ*relA::kan *was derived from the KEIO library clone JW2755 [2] and P1-transduced into TB82. Δ*spoT*::*kan *was introduced using AB1058) as donor strain for P1 transduction.

To measure activity of the promoters P_ara_, P_rsd _and P_apt_, MG1655 and TB80 were transformed [37] with plasmids that contain transcriptional promoter-*gfp *fusions [29].

### Microscopy

LB agar pads were prepared by filling a cavity of a sterile microscope cavity slide with a drop of freshly melted LB agar, and covering it with a cover slip to attain a flat surface. The cavity slide was transferred to a fridge for a short time to allow the agar to solidify. Upon removal of the cover slip and removal of excess LB agar, the pads can be inoculated with precultured cells as described in the next paragraph.

TB80 and TB84 were cultured over night at 37° in LB medium with 0.1% L-arabinose and diluted 1:100 into fresh LB medium containing 0.01% L-arabinose. In early exponential phase, cultures were washed at least twice in LB supplemented with 0.4% glucose to remove residual L-arabinose. Wildtype *E. coli *MG1655 was treated similar for control experiments. 1.5 μl of a washed and diluted culture were transferred to the surface of a pad of LB agar (supplemented with D-glucose, L-arabinose, chloramphenicol or kanamycin as indicated for individual experiments) in a microscope cavity slide. The agar pad was closed with a cover slip and sealed with vacuum grease. Under these conditions, cells can grow exponentially in a two-dimensional plane for many generations without restrictions [23]. The slide was mounted onto an automated microscope (Olympus BX81) and incubated at 37°C (Cube and Box incubation system, Life Imaging Services, Reinach, Switzerland). Images were recorded every 2 or 4 minutes. Intensity and exposure times to fluorescent light were minimized to avoid cellular damage. The resulting image sequences were analyzed with the Matlab based script package "Schnitzcell" (kindly provided by Michael Elowitz, CalTech, USA [18]), and data was extracted with custom-made Matlab scripts (Table 1).

**Table 1:** List of strains and plasmids

Strain name	Relevant genotype	Source
DY330	W3110*□lacU169 gal490 cI857 (cro-bioA)*	[42]
MG1655	*F- lambda- ilvG*- *rfb*-50 *rph-1*	[43]
TB55	MG1655 *araC-kan-yabI*	This study
TB79	kan-*araC*-P_ara_-*ygjD*	This study
TB80	frt::*araC*-P_ara_-*ygjD*	This study
TB82	frt::*araC*-P_ara_-*ygjD *Δ*relA*::kan	This study
TB83	frt::araC-P_ara_-*ygjD *Δ*relA*::frt	This study
TB84	frt::araC-P_ara_-*ygjD *Δ*relA*::frt Δ*spoT*::kan	This study
FfH	kan-*araC*-P_ara_-*ffh*	This study
DnaT	kan-*araC*-P_ara_-*dnaT*	This study
FldA	kan-*araC*-P_ara_-*fldA*	This study
AB1058	Δ*spoT*::*kan *Δ*relA*::frt	This study
pCP20	*FLP+ *λ cI857^+ ^λ P_R _Rep^ts ^Amp^R ^Cam^R^	[39]

### Statistical analysis

To quantify associations between phenotypic traits, we used non-parametric correlation analysis (Spearman's rank correlation in PASW Statistics 18.0).

## Authors' contributions

TB designed and carried out the experiments; TB, AB and MA drafted the manuscript; MA developed the statistical test; RPM wrote extensions for Matlab. All authors read and approved the final manuscript.

## Supplementary Material

Additional File 1 **Movie 1. TB80 (ppGpp**^**+**^**) growing on LB agar with 0.1% L-arabinose**. 100 frames (one frame per two minutes) were compressed into 10 seconds. The scale bar is 5 μm in size (same in all movies hereafter). Click here for file

Additional File 2 **Movie 2: MG1655 growing on LB agar with 0.4% glucose**. 100 frames (one frame per two minutes) were compressed into 10 seconds. Click here for file

Additional File 3 **Figure S1: MG1655 expressing GFP from P**_**ara **_**shifted from LB arabinose 0.01% to LB glucose 0.4%**. This experiment was performed with the wild type strain MG1655 carrying a plasmid encoding a transcriptional fusion of *gfp *to P_ara _[29]. The strain was grown in 0.01% arabinose, analogously to the depletion experiments with TB80 and TB84, washed in LB supplemented with glucose and transferred onto an agar pad consisting of LB agar with 0.4% glucose. The level of GFP fluorescence decreased rapidly and approached the level of background fluorescence when cells reached generation 4. Click here for file

Additional File 4 **Movie 3. TB80 (ppGpp**^**+**^**) growing on LB agar with 0.4% glucose**. 150 frames (one frame per two minutes) were compressed into 15 seconds. This movie was used to extract the growth dynamics shown in Figure 2 and 3. Click here for file

Additional File 5 **Figure S2: Lineage trees of microcolonies of A) MG1655 growth and B) YgjD depletion**. After tracking of individual cells across recorded images with "Schnitzcell", the lineage structure of a microcolony can be derived. In such a lineage tree, the branch length corresponds to the time interval between divisions, and division events occur at branching points. The different colors depict the color code used for cells from different generations throughout all figures. The dots at the end of individual branches represent the time points where individual physiological measurements (cell size and fluorescent intensity) were derived from. Click here for file

Additional File 6 **Figure S3: Depletion of essential genes induces unique phenotypes**. Time-lapse experiments of cells depleting for *fldA*, *ffh *and *dnaT *(see Additional Files 7, 8 and 9 - movies 4, 5 and 6) were tracked, and the cell size at division over consecutive divisions was plotted. Click here for file

Additional File 7 **movie 4: Depletion of FldA from growing cells**. A P_ara_-*fldA *conditional lethal mutant was shifted from 0.1% arabinose to an agar pad with 0.4% glucose. FldA is essential for isoprenoid biosynthesis [44], and as the movie shows, depletion of FldA leads to lysis of cells. 80 frames (one frame per four minutes) were compressed into 8 seconds. Click here for file

Additional File 8 **movie 5: Depletion of Ffh from growing cells**. A P_ara_-*ffh *conditional lethal mutant was shifted from 0.1% arabinose to an agar pad with 0.4% glucose. Ffh protein is part of the signal recognition particle translocation system, that cotranslationaly sequesters proteins into or across the cytoplasmic membrane [45]. Depletion resulted in visible intracellular aggregates, followed by elongation and cell lysis. 120 frames (one frame per two minutes) were compressed into 12 seconds. Click here for file

Additional File 9 **movie 6: Depletion of DnaT from growing cells**. A P_ara_-*dnaT *conditional lethal mutant was shifted from 0.01% arabinose to a 0.4% glucose containing agar pad. Depletion resulted in filament formation, which is in agreement with "unbalanced" growth upon abrogation of DNA replication. *dnaT *(and the following gene *dnaC*) is part of the "primosome" and is crucial for initiation of DNA replication. 100 frames (one frame per four minutes) were compressed into 10 seconds. Click here for file

Additional File 10 **Figure S4: Effects of minimum inhibitory concentrations (MIC) of chloramphenicol and kanamycin on growth of *E. coli *MG1655**. Recorded image series of *E.coli *MG1655 growing on MIC concentrations of chloramphenicol (2.5 μg/ml) and kanamycin (5 μg/ml) (see Additional Files 11 and 12 - movies 7 and 8) were tracked, and the cell size over consecutive division was plotted. Click here for file

Additional File 11 **movie 7: Growth of *E. coli *MG1655 on 2.5 μg/ml chloramphenicol**. *E. coli *MG1655 was precultured in LB medium and transferred to an agar pad containing 2.5 μg/ml chloramphenicol. 100 frames (one frame per four minutes) were compressed into 10 seconds,. Click here for file

Additional File 12 **movie 8: Growth of *E. coli *MG1655 on 5 μg/ml kanamycin**. *E. coli *MG1655 was precultured in LB medium and transferred to an agar pad containing 5 μg/ml kanamycin. 60 frames (one frame per four minutes) were compressed into 6 seconds. Click here for file

Additional File 13 **Figure S5: Coupling of cell elongation rate and interval between division across multiple experiments**. The pattern observed in Figure 3 is repeatable and consistent across independent experiments. Non-parametric correlation analysis for the differences between sisters in these two traits was performed for seven independent microcolonies (YgjD depletion in TB80), and the median and the range of the correlation coefficients is reported; the median correlation coefficients are negative from generation 3 on, indicating a coupling between cell elongation rate and the interval between two divisions. Click here for file

Additional File 14 **Movie 9. TB84 (ppGpp**^**0**^**) growing on LB agar with 0.4% glucose**. 200 frames (one frame per two minutes) were compressed into 20 seconds. Click here for file

Additional File 15 **Figure S6: YgjD is also essential in absence of (p)ppGpp**. Data of cell numbers versus time from three independent experiments; each experiment is based on a microcolony that was initiated with a single cell of strain TB84 (ppGpp^0^), and grown in the presence of glucose, leading to YgjD depletion. Cell division terminates after about five to six divisions. Click here for file

Additional File 16 **Figure S7: Control movies of P**_**apt **_**and P**_**rsd **_**expression of TB80 grown with 0.1% L-arabinose**. Single cell measurements of cell elongation rate and GFP fluorescence of two strains with transcriptional reporters for P_apt _(**A and B**) and P_rsd _(**B and C**), analogous to Figure 5 in the main manuscript. Click here for file

Additional File 17 **Figure S8: DNA staining of cells with and without YgjD in TB80 (ppGpp**^**+**^**) and TB84 (ppGpp**^**0**^**)**. Cells were grown for two hours in liquid culture, and stained with 1 μg/ml DAPI (4',6-diamidino-2-phenylindole) to visualize DNA. Scale bars are 5 μm. **A) **TB80 grown with 0.1% arabinose to induce YgjD expression. **B) **TB80 grown with 0.4% glucose, leading to YgjD depletion. Cells are small, and the DNA stain occupies a large fraction of the cell area. **C) **TB84 grown with 0.4% glucose, leading to YgjD depletion. Cells are elongated, and the DNA stain only occupies a small fraction of the cell area. Click here for file
