# Supplementary figures and images for: Single-cell time-lapse analysis of depletion of the universally conserved essential protein YgjD
Source: BMC Microbiol. 2011 May 27;11:118. doi: 10.1186/1471-2180-11-118 (PMC3115834; doi:10.1186/1471-2180-11-118)

**Mean GFP fluorescence intensity**

**Generations**

- 1
- 2
- 3
- 4
- 5
- 6

50

40

30

20

10

0

-10

0

20

40

60

80

100

120

140

**Time (minutes)**

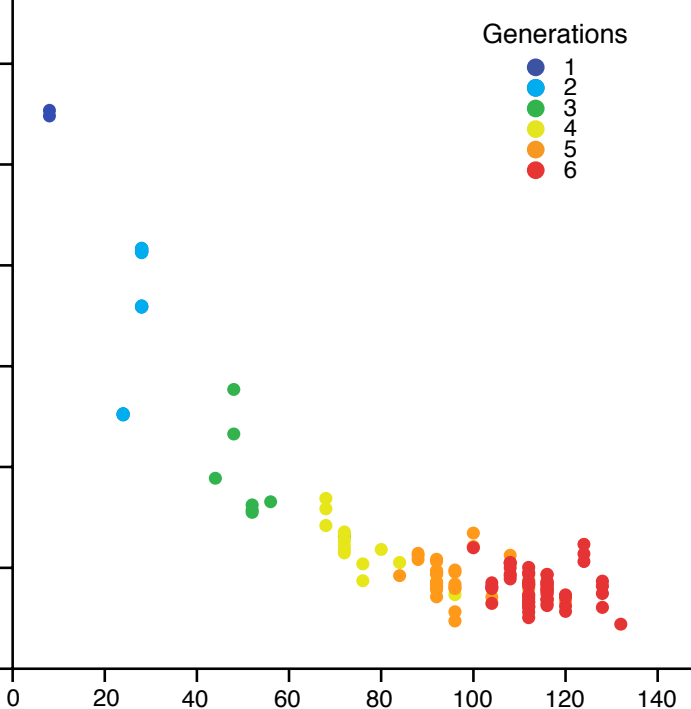

Supplement: Additional File 3 — Figure S1: MG1655 expressing GFP from Para shifted from LB arabinose 0.01% to LB glucose 0.4%. This experiment was performed with the wild type strain MG1655 carrying a plasmid encoding a transcriptional fusion of gfp to Para [29]. The strain was grown in 0.01% arabinose, analogously to the depletion experiments with TB80 and TB84, washed in LB supplemented with glucose and transferred onto an agar pad consisting of LB agar with 0.4% glucose. The level of GFP fluorescence decreased rapidly and approached the level of background fluorescence when cells reached generation 4. [file 1471-2180-11-118-S3.PDF]

**A**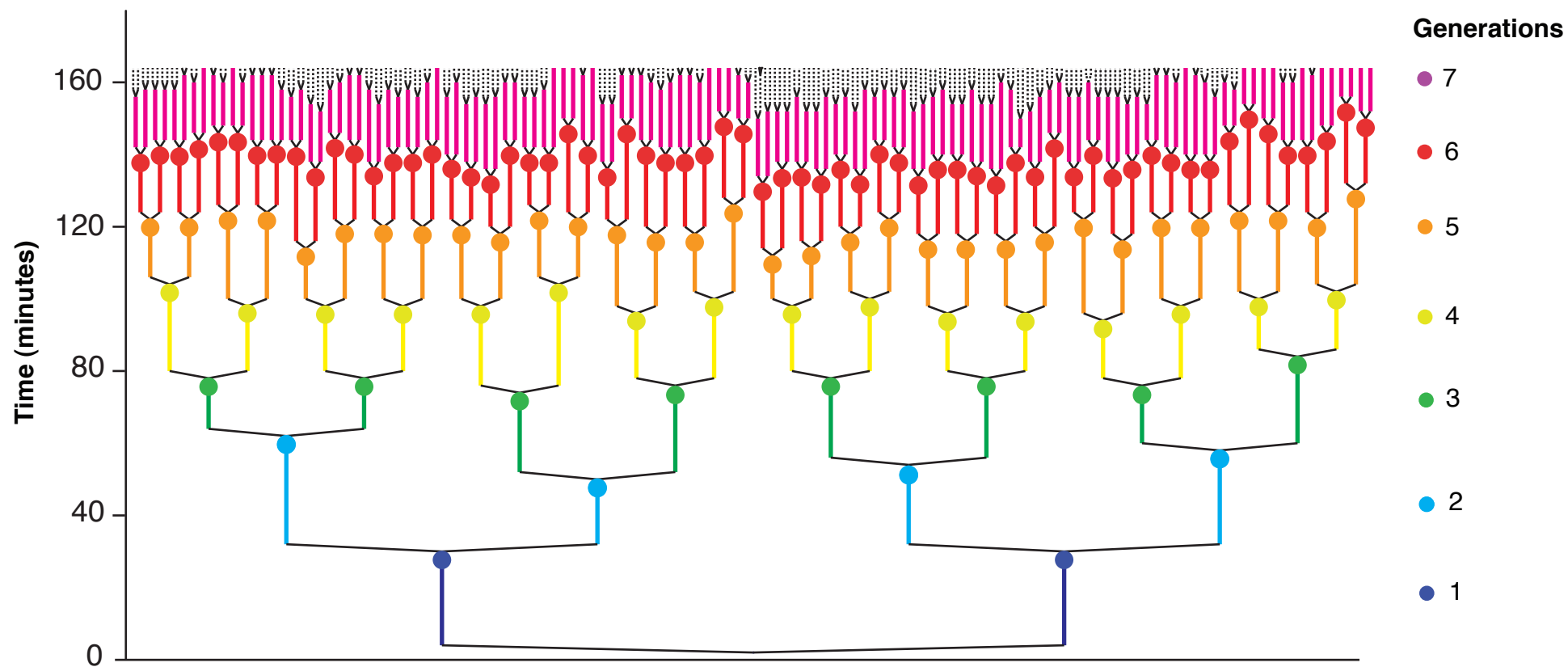**B**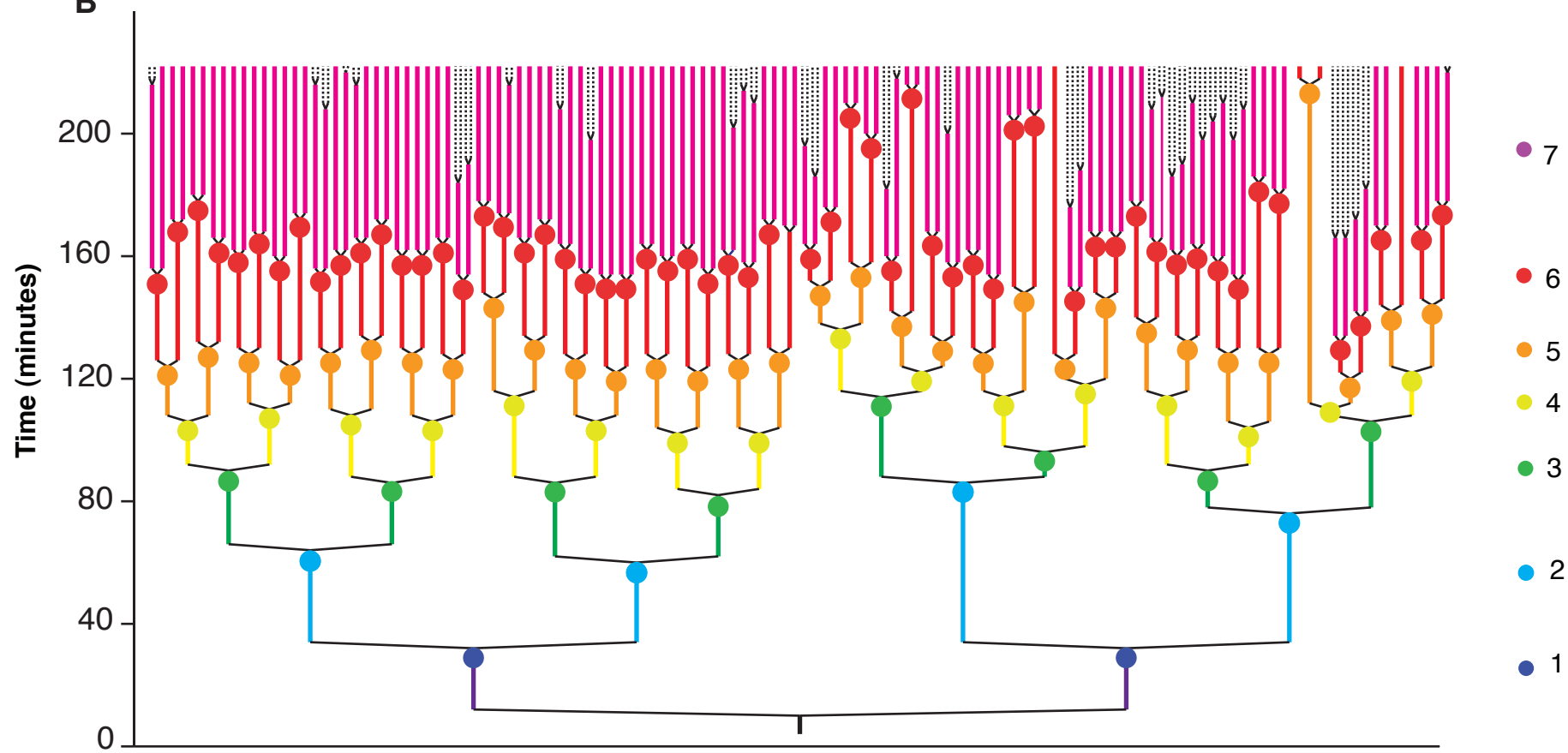

Supplement: Additional File 5 — Figure S2: Lineage trees of microcolonies of A) MG1655 growth and B) YgjD depletion. After tracking of individual cells across recorded images with "Schnitzcell", the lineage structure of a microcolony can be derived. In such a lineage tree, the branch length corresponds to the time interval between divisions, and division events occur at branching points. The different colors depict the color code used for cells from different generations throughout all figures. The dots at the end of individual branches represent the time points where individual physiological measurements (cell size and fluorescent intensity) were derived from. [file 1471-2180-11-118-S5.PDF]

**A** **fliA depletion**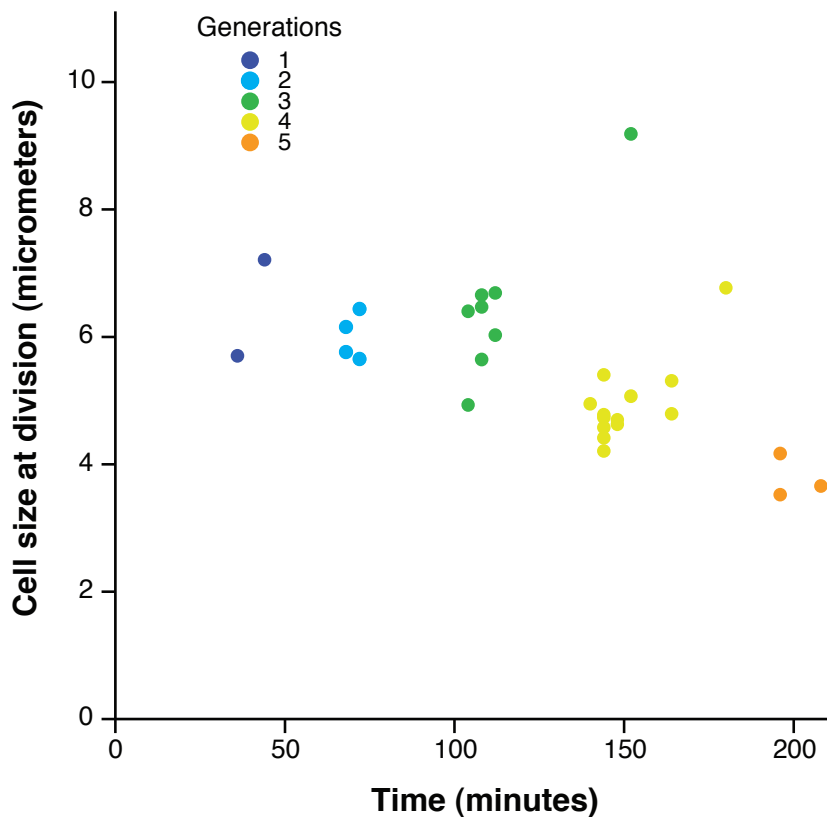**B** **ffh depletion**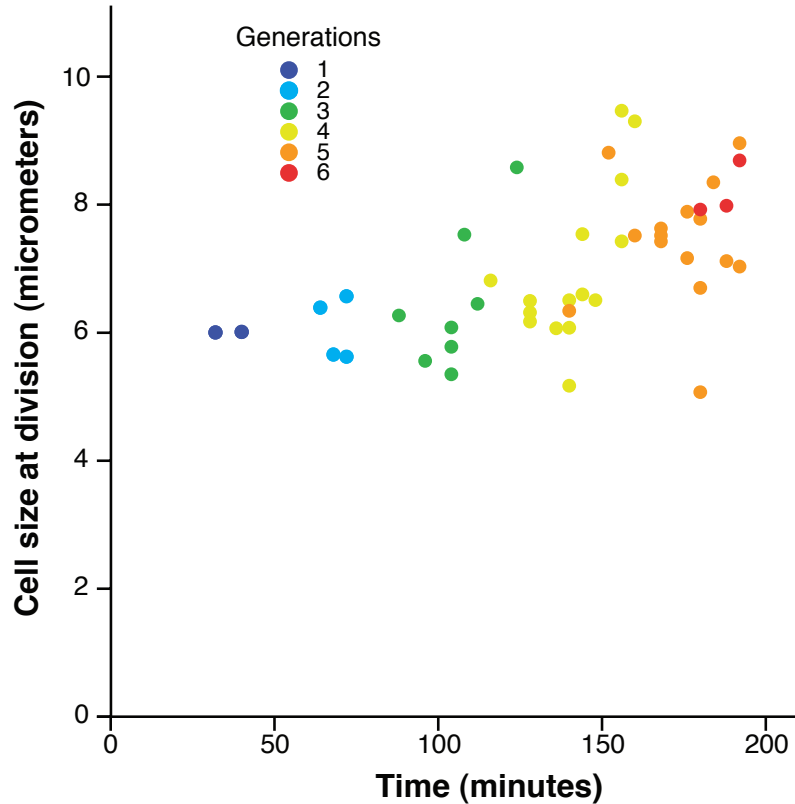**C** **dnaT depletion**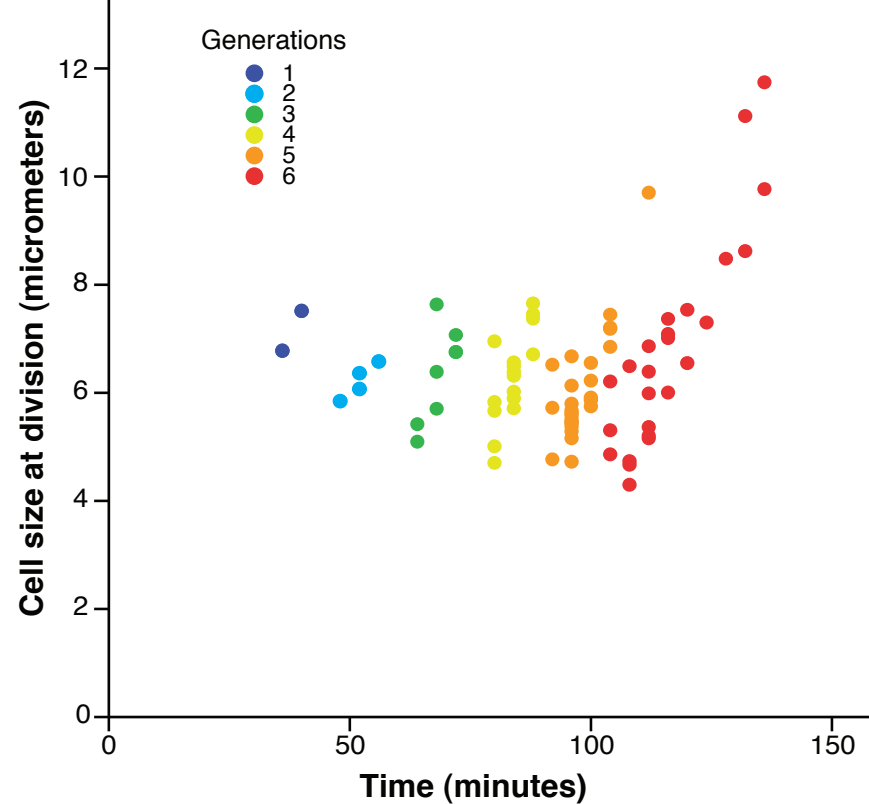

Supplement: Additional File 6 — Figure S3: Depletion of essential genes induces unique phenotypes. Time-lapse experiments of cells depleting for fldA, ffh and dnaT (see Additional Files 7, 8 and 9 - movies 4, 5 and 6) were tracked, and the cell size at division over consecutive divisions was plotted. [file 1471-2180-11-118-S6.PDF]

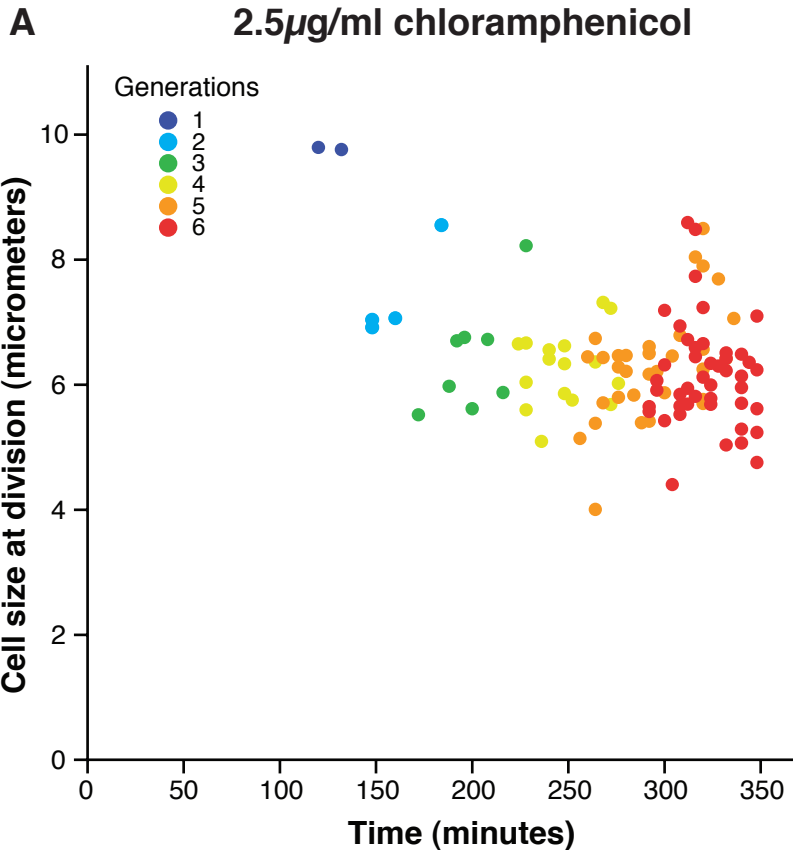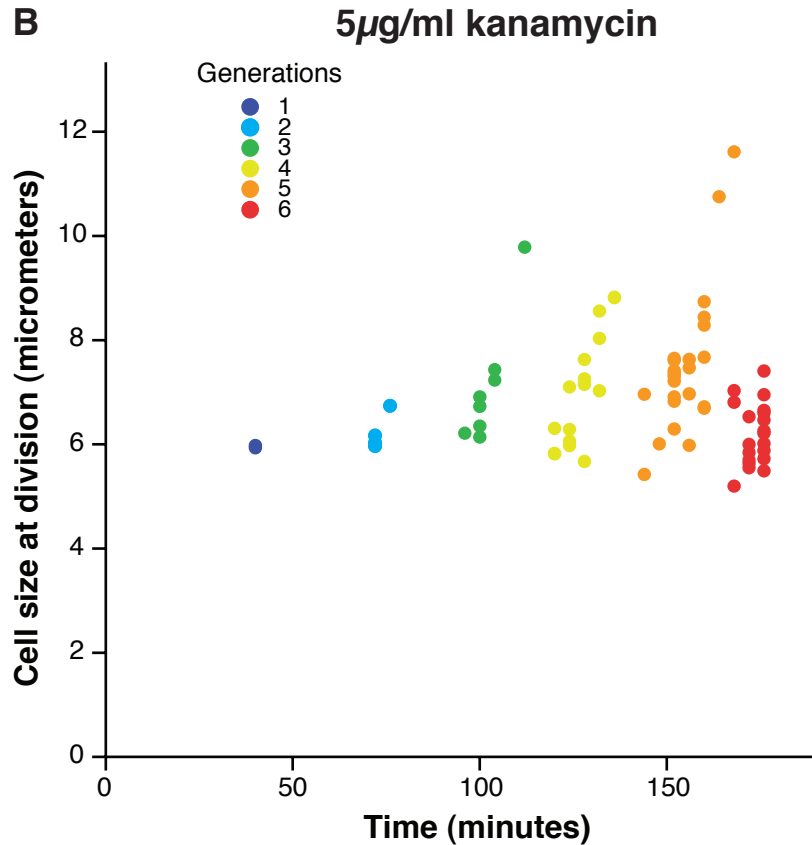

Supplement: Additional File 10 — Figure S4: Effects of minimum inhibitory concentrations (MIC) of chloramphenicol and kanamycin on growth of E. coli MG1655. Recorded image series of E.coli MG1655 growing on MIC concentrations of chloramphenicol (2.5 μg/ml) and kanamycin (5 μg/ml) (see Additional Files 11 and 12 - movies 7 and 8) were tracked, and the cell size over consecutive division was plotted. [file 1471-2180-11-118-S10.PDF]

Median Spearman's rho

Generations

- 3
- 4
- 5
- 6

0.8  
0.6  
0.4  
0.2  
0  
-0.2  
-0.4  
-0.6  
-0.8  
-1.0

3

4

5

6

Generations

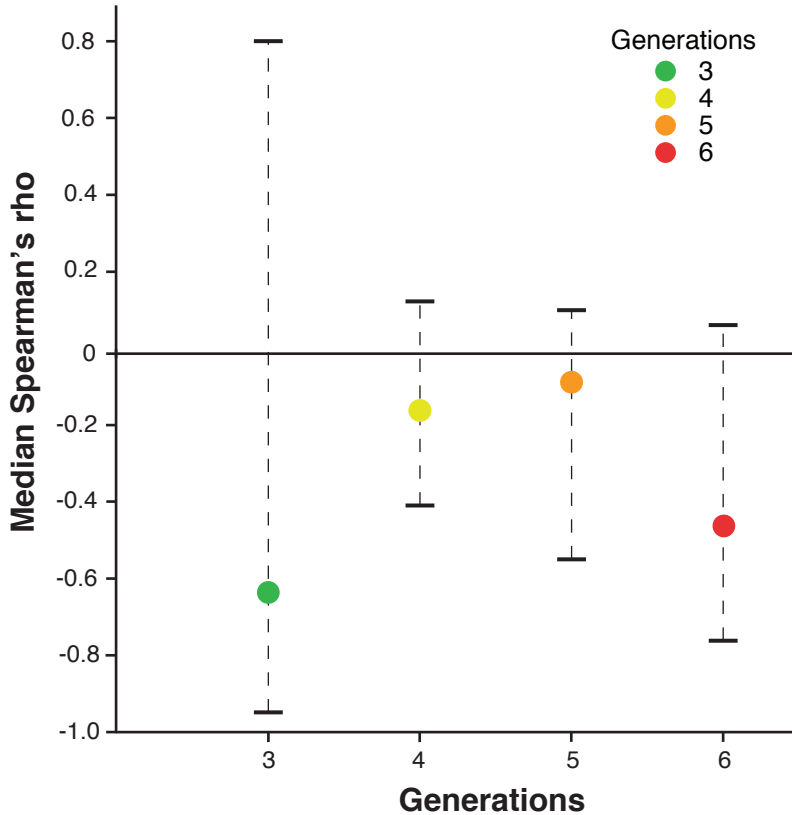

Supplement: Additional File 13 — Figure S5: Coupling of cell elongation rate and interval between division across multiple experiments. The pattern observed in Figure 3 is repeatable and consistent across independent experiments. Non-parametric correlation analysis for the differences between sisters in these two traits was performed for seven independent microcolonies (YgjD depletion in TB80), and the median and the range of the correlation coefficients is reported; the median correlation coefficients are negative from generation 3 on, indicating a coupling between cell elongation rate and the interval between two divisions. [file 1471-2180-11-118-S13.PDF]

Number of cells

0

50

100

150

200

250

Time (minutes)

100

10

1

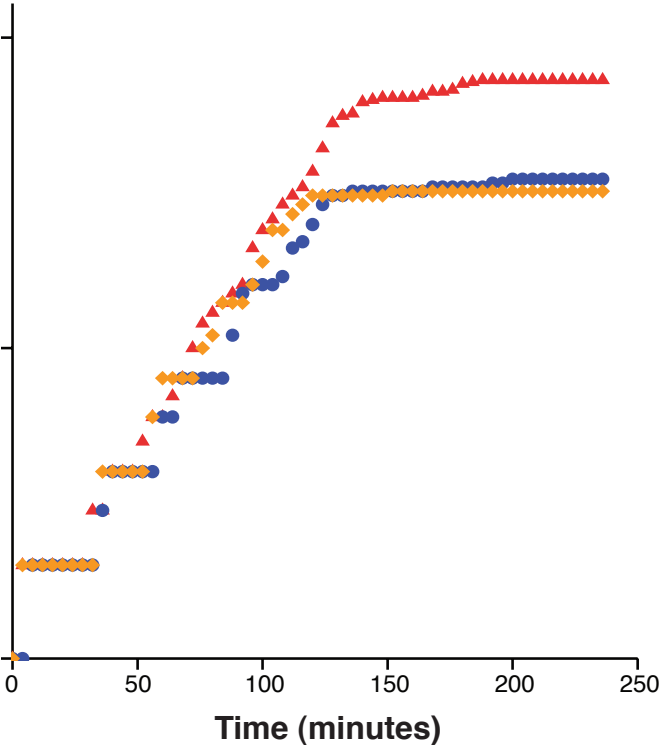

Supplement: Additional File 15 — Figure S6: YgjD is also essential in absence of (p)ppGpp. Data of cell numbers versus time from three independent experiments; each experiment is based on a microcolony that was initiated with a single cell of strain TB84 (ppGpp0), and grown in the presence of glucose, leading to YgjD depletion. Cell division terminates after about five to six divisions. [file 1471-2180-11-118-S15.PDF]

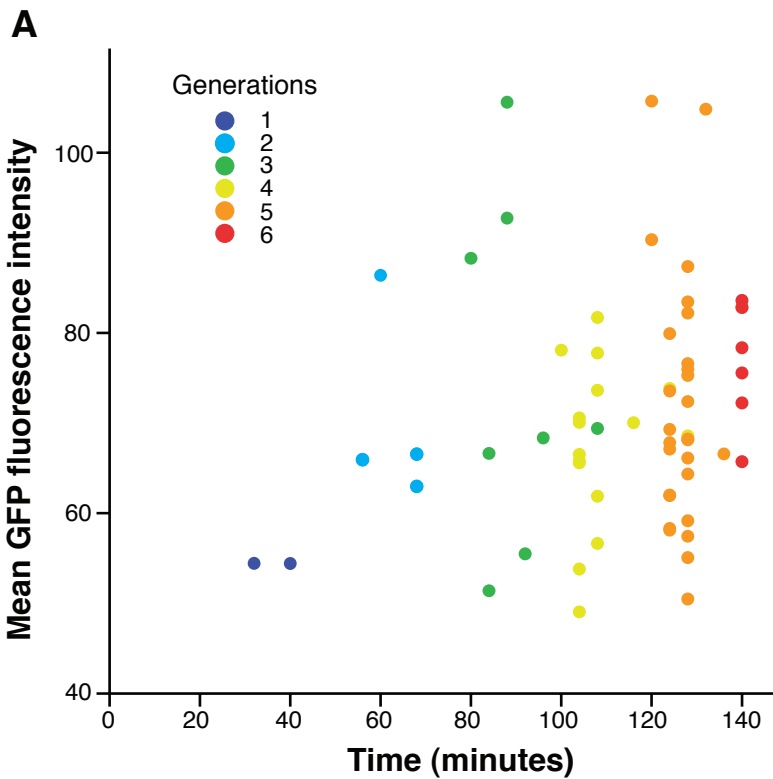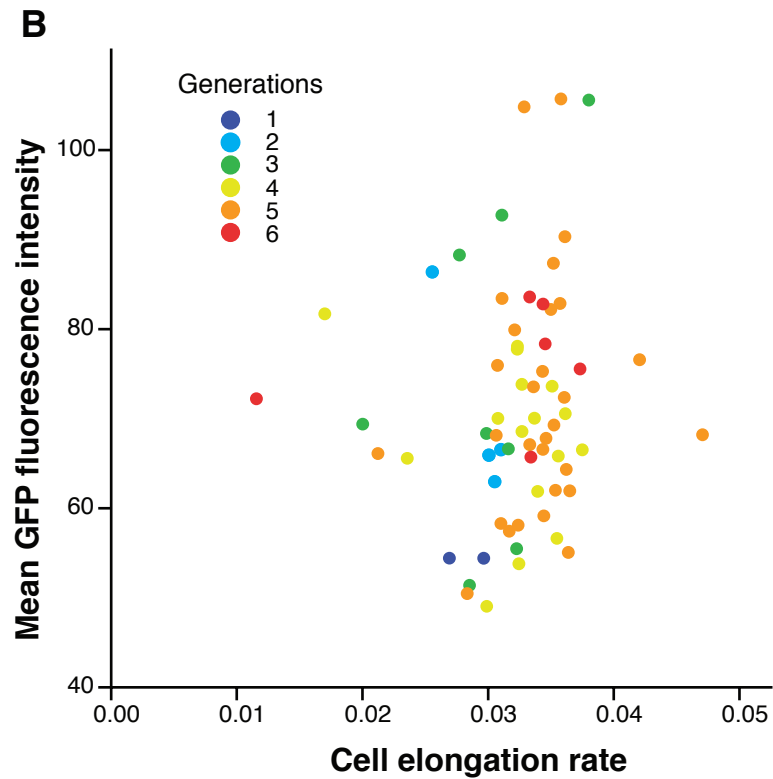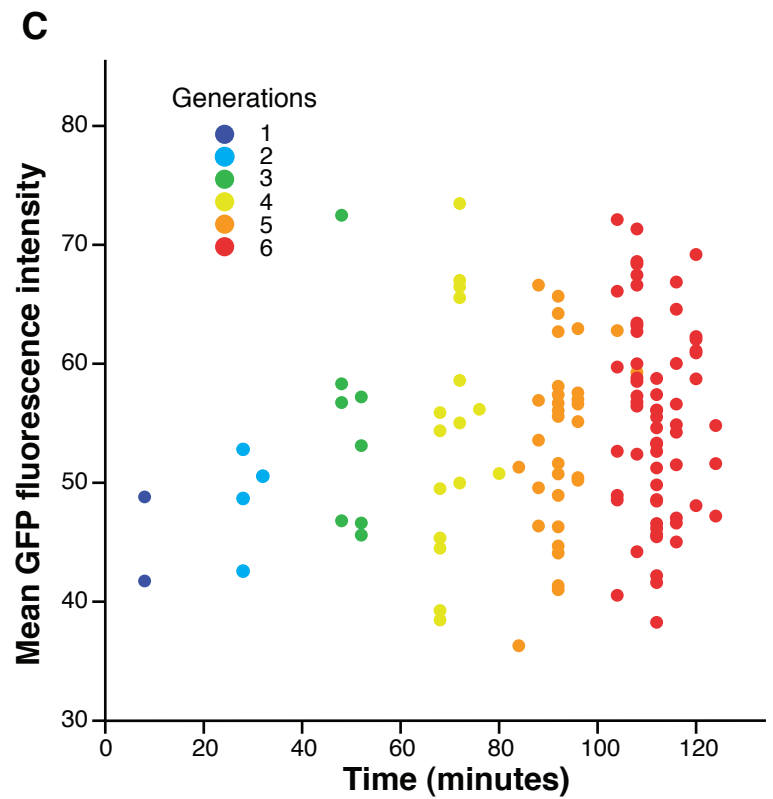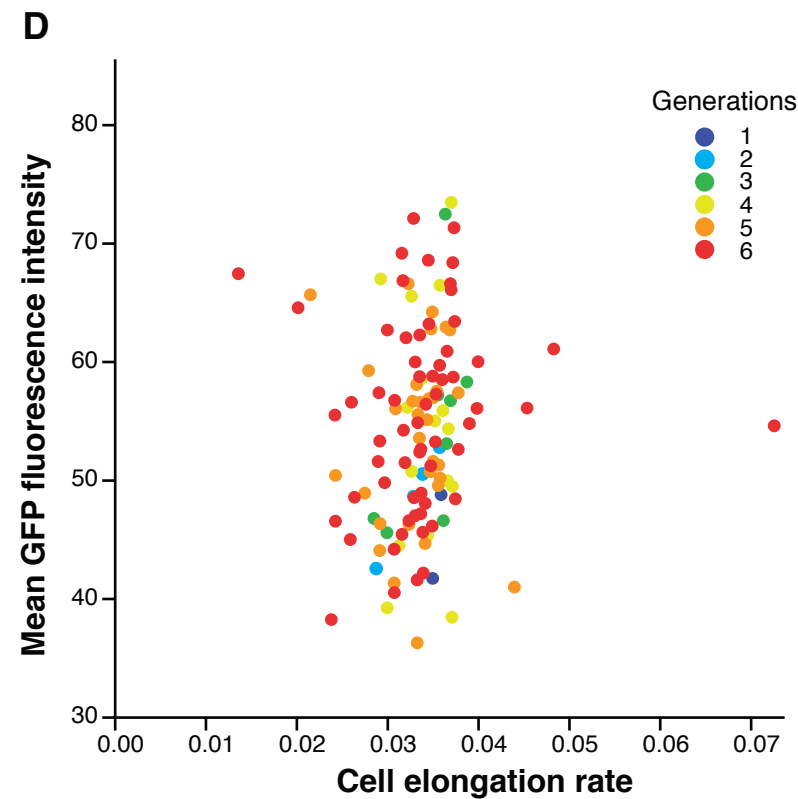

Supplement: Additional File 16 — Figure S7: Control movies of Papt and Prsd expression of TB80 grown with 0.1% L-arabinose. Single cell measurements of cell elongation rate and GFP fluorescence of two strains with transcriptional reporters for Papt (A and B) and Prsd (B and C), analogous to Figure 5 in the main manuscript. [file 1471-2180-11-118-S16.PDF]

**A**

Phase contrast

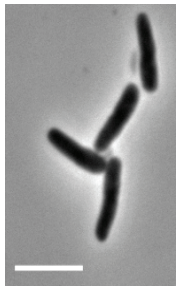

DAPI

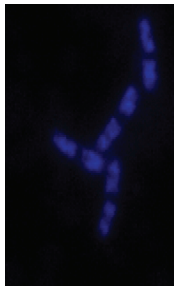

Merge

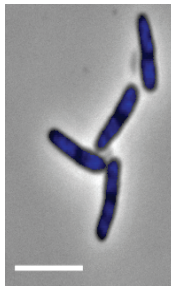**B**

Phase contrast

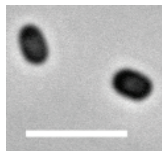

DAPI

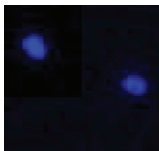

Merge

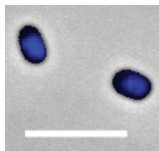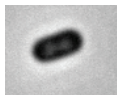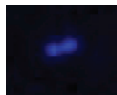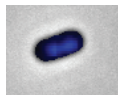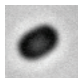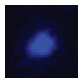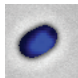**C**

Phase contrast

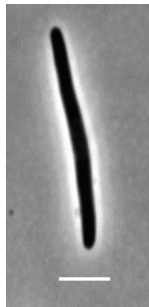

DAPI

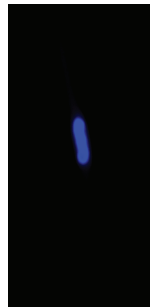

Merge

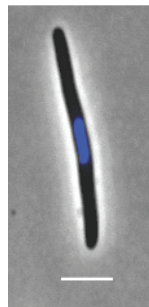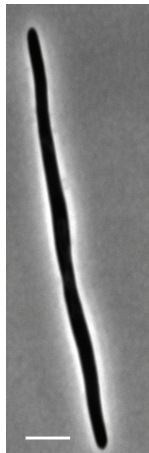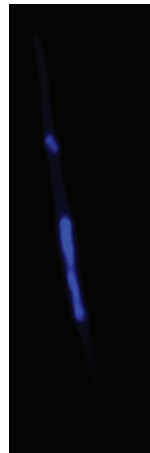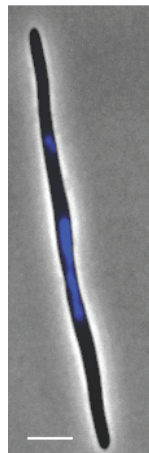

Supplement: Additional File 17 — Figure S8: DNA staining of cells with and without YgjD in TB80 (ppGpp+) and TB84 (ppGpp0). Cells were grown for two hours in liquid culture, and stained with 1 μg/ml DAPI (4',6-diamidino-2-phenylindole) to visualize DNA. Scale bars are 5 μm. A) TB80 grown with 0.1% arabinose to induce YgjD expression. B) TB80 grown with 0.4% glucose, leading to YgjD depletion. Cells are small, and the DNA stain occupies a large fraction of the cell area. C) TB84 grown with 0.4% glucose, leading to YgjD depletion. Cells are elongated, and the DNA stain only occupies a small fraction of the cell area. [file 1471-2180-11-118-S17.PDF]
